# *Zkscan3* affects erythroblast development by regulating the transcriptional activity of GATA1 and KLF1 in mice

**DOI:** 10.1007/s10735-021-10052-8

**Published:** 2021-12-23

**Authors:** Zixuan Li, Binjie Sheng, Tingting Zhang, Tian Wang, Dan Chen, Gangli An, Xingbing Wang, Huimin Meng, Lin Yang

**Affiliations:** 1 State Key Laboratory of Radiation Medicine and Protection, Cyrus Tang Medical Institute, Collaborative Innovation Center of Hematology, Soochow University, Suzhou, 215123 China; 2 Division of Life Sciences and Medicine, Department of Hematology, The First Affiliated Hospital of USTC, University of Science and Technology of China, Hefei, 230001 Anhui People’s Republic of China; 3 Cyrus Tang Hematology Center, Soochow University, Suzhou, Jiangsu China

**Keywords:** zkscan3, Erythroblast development, GATA1, KLF1, Mouse

## Abstract

*ZKSCAN3* encodes a zinc-finger transcription factor that regulates the expression of important genes and plays a significant role in tumor development, pathogenesis, and metastasis. However, its biological functions under normal physiological conditions remain largely unknown. In our previous studies, using flow cytometry, we found that the deletion of *Zkscan3* may cause abnormal erythropoiesis. In this study, we found that, in a *Zkscan3* knockout mice model, the number of splenic early-stage (basophilic-erythroblasts) and late-stage (chromatophilic-erythroblasts to polychromatophilic-erythroblasts through orthochromatophilic-erythroblasts) erythroblasts increased, whereas the number of late erythroblasts in the bone marrow decreased. Moreover, the phenotype was exacerbated after treating mice with phenylhydrazine (PHZ), which causes severe hemolytic anemia. In the knockout mice treated with PHZ, the percentage of reticulocyte in the peripheral blood conspicuously increased, whereas MCHC and red blood cells decreased. Then, we performed RNA-seq and quantitative-polymerase chain reaction assay and found that the expression of *GATA1* and *Tiam1* in erythroblasts were upregulated, whereas *KLF1* was downregulated. Luciferase assays showed that Zkscan3 inhibited the transcription of *GATA1* and *Tiam1* and promoted the expression of *KLF1*. Additionally, ChIP and CO-IP results confirmed that Zkscan3 directly interacts with GATA1 and inhibits its transcriptional activity in MEL cells. Our results demonstrate, for the first time, the significant role of Zkscan3 in physiological erythropoiesis through the interaction with GATA1, both at the DNA and protein level, and with *Tiam1* and *KLF1* at the DNA level.

## Introduction

ZKSCAN3, a zinc finger protein containing the KRAB and SCAN 3 domains, is a member of the Krüppel-associated box domain zinc finger protein (KRAB-ZFP) family, the largest family of transcriptional regulators. Members of this family are involved in multiple cellular processes, including cell proliferation, apoptosis, and neoplastic transformation (Urrutia 2003; Ecco et al. 2017). KRAB-ZFP has four subfamilies, two comprising protein with KRAB and C2H2 zinc finger motifs, and the other two comprising an additional SCAN (SCAN-ZFP) domain at the N-terminus. The KRAB domain has a transcriptional inhibition function, while the C2H2 zinc finger domain confers binding specificity to a particular DNA sequence. However, the SCAN domain function is still poorly understood (Urrutia 2003; Ecco et al. 2017). Zkscan3 and its gene, *Zkscan3*, has been reported to promote cancer cell proliferation, migration, and invasion by upregulating the expression of genes associated with cell cycle, cell proliferation, migration, angiogenesis, and proteolysis (Yang et al. 2008, 2011; Zhang et al. 2012; Chi et al. 2018; Lee et al. 2018). However, a comprehensive description of the *Zkscan3* function, particularly in physiological conditions, is still lacking. Previously, we established a novel *Zkscan3* knockout mice model and observed the presence of splenic lesions and heme-like deposits (Zixuan et al. 2019). Therefore, we speculated that the erythroblast development may be abnormal in the absence of *Zkscan3*.

In mammals, erythropoiesis mostly occurs in the bone marrow. The terminal steps involve the expulsion of the erythroblast nucleus, leading to the formation of reticulocytes. This process involves complex biological processes, such as cell membrane remodeling, rearrangement of the actin cytoskeleton, chromatin condensation, and nuclear polarization (Moras et al. 2017). Various stress factors can impede erythrocyte development and function, causing anemia. Despite its importance, our knowledge of the underlying cellular and molecular mechanisms in erythrocyte maturation is incomplete. Erythroblast stages are commonly classified using fluorescence activated cell sorting analysis (FACS) based on the expression of glycophorin A (TER119) and the transferring receptor (CD71). Erythroid cells are sorted into 4 populations: preliminary stage (pro-erythroblasts; TER119^middle^/CD71^high^), early-stage (basophilic-erythroblasts; TER119^high^/CD71^high^), late-stage (chromatophilic-erythroblasts to polychromatophilic-erythroblasts through orthochromatophilic-erythroblasts; TER119^high^/CD71^middle^), and mature stage (reticulocytes; TER119^high^/CD71^low^) (Zhang et al. 2003; Rivkin et al. 2017).

GATA binding factor 1 (GATA1) is a zinc-finger transcription factor that plays a critical role in regulating erythroid development (Cantor and Orkin 2002). Studies using animal and in vitro differentiation models have shown that the dysregulation of GATA1 causes failed erythropoiesis. *GATA1* mutations are associated with congenital erythroid hypoplasia and X-linked abnormal dyserythropoietic anemia (Crispino and Horwitz 2017). Within these cell lineages, GATA1 regulates the expression of a plethora of genes and its function depends by its ability to bind both the DNA and different protein partners, including itself (c-Myb, EKLF, Fli-1, FOG, HDAC5, LMO2, PU.1, RUNX1, CREB-binding protein/P300, Ski-1, and Sp1, among others) (Lowry and Mackay 2006; DeVilbiss et al. 2016). KLF1, a C2H2 zinc finger protein, is another essential factor for the enucleation of erythroid precursors in the late-stages of erythrocyte differentiation (Gnanapragasam et al. 2016). In the literature, the interaction of Zkscan3 with either GATA1 or KLF1 has not been reported.

Here, we report the effect of *Zkscan3* knockout on erythrocyte maturation and the interaction of Zkscan3 with GATA1 and KLF1.

## Materials and methods

### Antibodies

The following antibodies were used in this study: anti-CD71-PE (cat:113808, Biolegend, San Diego, CA), anti-Ter119-APC (cat:116223, Biolegend), anti-7-AAD Staining Solution (cat:559925, BD Biosciences, San Jose, CA),anti-GFP (4B10) Mouse mAb (cat:2955, Cell Signaling Technology, Danvers, MA), Donkey anti-Rat Alexa Fluor 594 (cat: A48271, Thermo Fisher Scientific, Waltham, MA), purified rat anti-mouse CD71 (cat:113802, Biolegend), FITC anti-Ter119 (cat: 11-5921-82, eBioscience), anti-GATA-1 (D52H6) XP Rabbit mAb (cat:3535, Cell Signaling Technology), anti-GAPDH (cat:100118,Gene Tex, Irvine, CA, USA), anti-rabbit lgG (cat:HAF008, Cell Signaling Technology), anti-His-Tag (D3I1O) XP Rabbit mAb (cat:12698, Cell Signaling Technology).

### Flow cytometry

The spleen and bone marrow cells collected from 6 to 8-weeks-old mice were stained with anti-CD71-PE (1:100) and anti-Ter119-APC (1:100) antibodies for 20 min at 4 °C in the dark. Then were stained with 7-AAD to aid dead cell exclusion for accurate live cell analysis. Erythroblast staining was performed as previously described (Zhang et al. 2003; Rivkin et al. 2017). Flow cytometric analyses were performed using Gallios Flow Cytometer (Beckman Coulter, Franklin Lakes, NJ, USA).

### Immunofluorescence

Slices (10 µM) of fresh spleen tissue were fixed with 4% paraformaldehyde for 10 min and blocked with 5% bovine serum album in phosphate-buffered saline for 1 h at room temperature and incubated with purified rat anti-mouse CD71 (1:100) overnight at 4 °C. The slices were incubated with donkey anti-rat Alexa Fluor 594 (1:500) for 2 h at room temperature, washed three times with phosphate-buffered saline, and incubated with FITC anti-Ter119 (1:200) for 2 h at room temperature. Images were acquired using a confocal fluorescence microscope (FV3000; Olympus, Tokyo, Japan).

### Mice

All *Zkscan3*^−/−^ animals and wild-type controls were the progeny of the same colony of C57BL/6 mice. *Zkscan3*^−/−^ animals model generation and genotyping were described previously (Zixuan et al. 2019). All animal experiments were approved by the Ethics Committee of Suzhou University. Animal welfare and national regulations for the management and ethics of animal experimentation were extensively adhered to for the experimental design and the implementation of our study (approval ID NSFC2014:31471283).

### Phenylhydrazine treatment

Phenylhydrazine (PHZ) treatment was performed as previously reported (Liao et al. 2018). Briefly, mice were injected intraperitoneally (i.p.) with PHZ at a dose of 100 mg/kg body weight. Erythroblast subsets in the spleen and bone marrow samples were analyzed on days 3 and 10 after PHZ treatment by flow cytometry (*n* = 7–8/group).

### Quantitative polymerase chain reaction (qPCR)

The early-stage erythroblasts were sorted by flow cytometry using a FACSAria III system (BD Biosciences, Franklin Lakes, NJ, USA) (n = 4/group). RNA was isolated from freshly sorted the early-stage erythroblast using the MicroElute Total RNA Kit (R6831; Omega Bio-tek, Norcross, GA, USA) (*n* = 4). cDNA was generated by reverse transcription of 0.2 µg of total RNA using the RevertAid First Strand cDNA Synthesis Kit (k1622; Thermo Fisher Scientific, Waltham, MA, USA) in a total reaction volume of 20 µL, following the manufacturer’s instructions. qPCR was performed on a High Throughput Quantitative PCR apparatus (LightCycler 480 II, Roche Applied Science, Basel, Switzerland) using the SYBR Green Premix Ex Taq PCR Array kit (RR420A TaKaRa, Shiga, Japan), following the manufacturer’s instruction. The following primers were used: *GATA1* (TGGGGACCTCAGAACCCTTG and GGCTGCATTTGGGGAAGTG), *Tiam1* (GAAGCACACTTCACGCTCC and CTCCAGGCCATTTTCAGCCA), *GAPDH* (AGGTCGGTGTGAACGGATTTG and TGTAGACCATGTAGTTGAGGTCA), and *KLF1* (AGACTGTCTTACCCTCCATCAG and GGTCCTCCGATTTCAGACTCAC). Quantification was determined by 2^−ΔΔCt^ method, and transcript levels of target genes were normalized with *GAPDH*.

### Luciferase assay

First, we constructed Zkscan3 fusion insert with His and GFP tags (Zkscan3-his-GFP). CT26 and mouse erythroleukemia (MEL) cells were transfected with lentivirus carrying this insert to obtain CT26-ZK3 and MEL-ZK3 cell lines overexpressing Zkscan3. Wild-type CT26 cells and CT26 cells overexpressing Zkscan3 gene (CT26-ZK3) were maintained in 1640 medium containing 10% fetal bovine serum. For transient transfection, cells were plated into six-well plates and transfected using lipofectamine 2000 according to the manufacturer’s instructions (Invitrogen, Carlsbad, CA, USA). The GATA1 reporter vector contained the firefly luciferase gene under the control of the –1000–0 bp promoter. The Tiam1 reporter vector contained the firefly luciferase gene under the control of the − 500–0 bp Tiam1 promoter. The KLF1 reporter vector contained the firefly luciferase gene under the control of the − 1000– + 150 bp KLF1 promoter. The total amount of DNA per transfection was normalized using an empty vector (pGL4.17). Luciferase activity was determined 48 h after transfection using a dual-luciferase assay kit (Promega, Madison, WI, USA) and measured with a Microplate Luminometer (Thermo Fisher Scientific).

### Co-immunoprecipitation

MEL cells were transfected with plasmids expressing Zkscan3 fused with a His-tag and GFP (Zkscan3-his-GFP), harvested, and lysed. The supernatants were collected and diluted to a concentration of 1 mg/mL. A part of the protein was used as input control, and the remaining protein was mixed with Ni–NTA agarose and incubated in a rotation plate overnight at 4 °C. Then, supernatants were centrifuged at 6000 rpm for 2 min at 4 °C. In western blot analysis, sole empty Ni–NTA agarose beads were used as a negative control. The beads were washed with protein lysing buffer six times. After centrifugation, the supernatant was collected, mixed with 5 × SDS loading buffer, and denatured at 95 °C on a thermomixer for 10 min in preparation for western blot analysis.

### Chromatin immunoprecipitation

We performed chromatin immunoprecipitation (ChIP) using the Simple ChIP Plus Sonication Chromatin IP Kit. (Catalog number 56383, Cell Signaling Technology), according to the manufacturer’s instructions. Briefly, protein-DNA complexes were cross-linked with 1% formaldehyde and the reaction was quenched by adding glycine (final concentration: 200 mM). The chromatin complexes were sonicated to generate 400–600 bp fragments using SCNICS VCX130. For immunoprecipitation, 5 µg DNA was diluted with chip buffer to make the volume to 500 µL and 2% of the total volume is used as the input. The ChIP assay was performed overnight at 4 °C using anti-His antibody (Cell Signaling Technologies), positive control antibody (Histone H3 [D2B12] XP Rabbit mAb), and negative control antibody (Normal Rabbit IgG antibody). The cross-linked DNA–protein complex was washed and eluted with 1X ChIP Buffer (10% 10 × ChIP Buffer + 90% H_2_O) at 4 °C for 5 min and the process was repeated three times. The complex was again diluted in 1X ChIP Buffer (10% 10 × ChIP Buffer + 90% H2O containing 7% 5 M NaCl) at 4 °C for 5 min. The bound fraction was isolated using protein G beads according to the manufacturer’s instructions, and the immunocomplexes were subjected to reverse cross-linking. The immunoprecipitated DNA was recovered using a PCR purification kit according to the manufacturer’s instructions, and the purified DNA was subjected to real-time quantitative PCR for further analysis (The primers for the positive control were provided in the kit).

### RNA-sequence

We sorted the early-stage erythroblast in the mouse spleen (*n* = 3), extracted RNA, and sent them to GENEWIZ (China) for quality inspection and RNA-sequence bioinformatics analysis.

### Generation of Zkscan3-KO mice

**Fig. 1:**
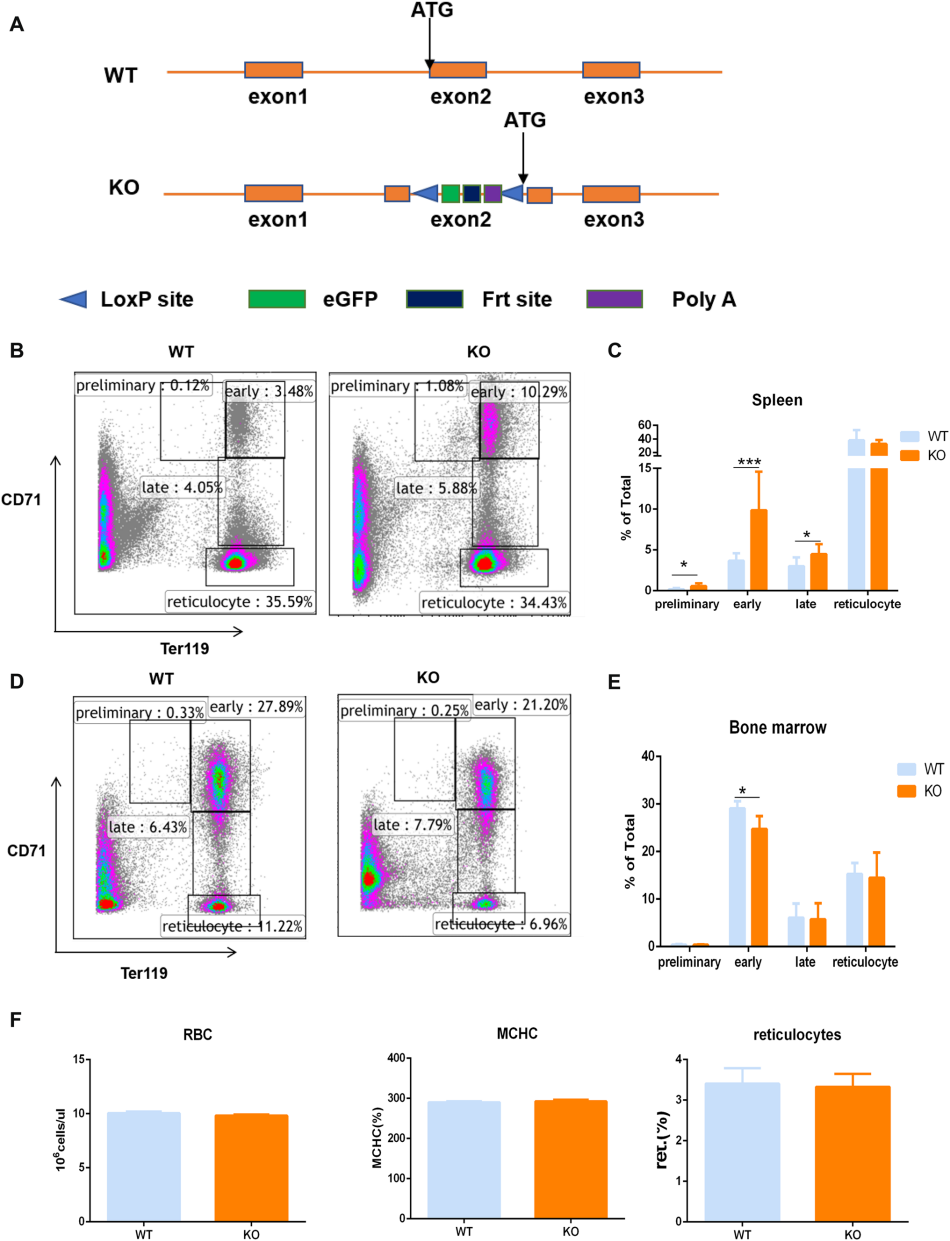
Erythroblast maturation in *Zkscan3* knockout mice and wild-type mice. **A** Schematic diagram of Zkscan3 knockout model. The enhanced green fluorescent protein (eGFP) expression cassette and terminator were inserted upstream of the start codon of Zkscan3. As shown in (a), the targeting vector was constructed by inserting an eGFP fragment and polyA terminator upstream of the start codon of Zkscan3, while Neo flanked by Frt sites was inserted downstream of the polyA sequence. In addition, LoxP sites were inserted upstream and downstream in the same orientation from the entire insertion cassette. The target sequence was inserted into the genomic DNA of ES cells via homologous recombination between the vector and genomic DNA. Next, positive ES cell clones were screened based on the resistance conferred by Neo and were implanted into surrogate mice, resulting in the development of chimeric mice carrying the target sequence. **B**–**E** We chose 6–8 weeks old wild-type and knockout mice to analyze the number of erythroblasts at each stage using flow cytometry. Stages were determined based on Ter119 and CD71 expression. The percentage of cells in each stage is indicated (*n* = 8, replicates per group, non-parametric statistical and Student’s *t* tests). **F** Analysis of RBC, MCHC and reticulocytes in the peripheral blood using the five-class blood corpuscle analyzer (*n* = 8, replicates per group, non-parametric statistical and Student’s *t* tests). (***p < 0.001, **p < 0.01, *p < 0.05)

The enhanced green fluorescent protein (eGFP) expression cassette and terminator were inserted upstream of the start codon of *Zkscan3*. In addition, Neo was inserted to facilitate the screening of cloned embryonic stem (ES) cells. The Frt sites flanking Neo were present to remove Neo after constructing the KO C57BL/6 mice to prevent phenotypic changes in mice resulting from its expression. Both the upstream and downstream LoxP sequences can be used to restore Zkscan3 expression when necessary. As shown in Fig. 1A, the targeting vector was constructed by inserting an eGFP fragment and polyA terminator upstream of the start codon of *Zkscan3*, while Neo flanked by Frt sites was inserted downstream of the polyA sequence. In addition, LoxP sites were inserted upstream and downstream in the same orientation from the entire insertion cassette. The target sequence was inserted into the genomic DNA of ES cells via homologous recombination between the vector and genomic DNA. Next, positive ES cell clones were screened based on the resistance conferred by Neo and were implanted into surrogate mice, resulting in the development of chimeric mice carrying the target sequence.

### Statistical analysis

Statistical analyses were performed using Mann–Whitney, Kruskal Wallis, and Student’s *t* tests through Prism 6.01 (GraphPad Software, San Diego, CA, USA). P value < 0.05 was considered statistically significant.

## Results

### Effect of *Zkscan3* knockout in erythroblast differentiation

**Fig. 2:**
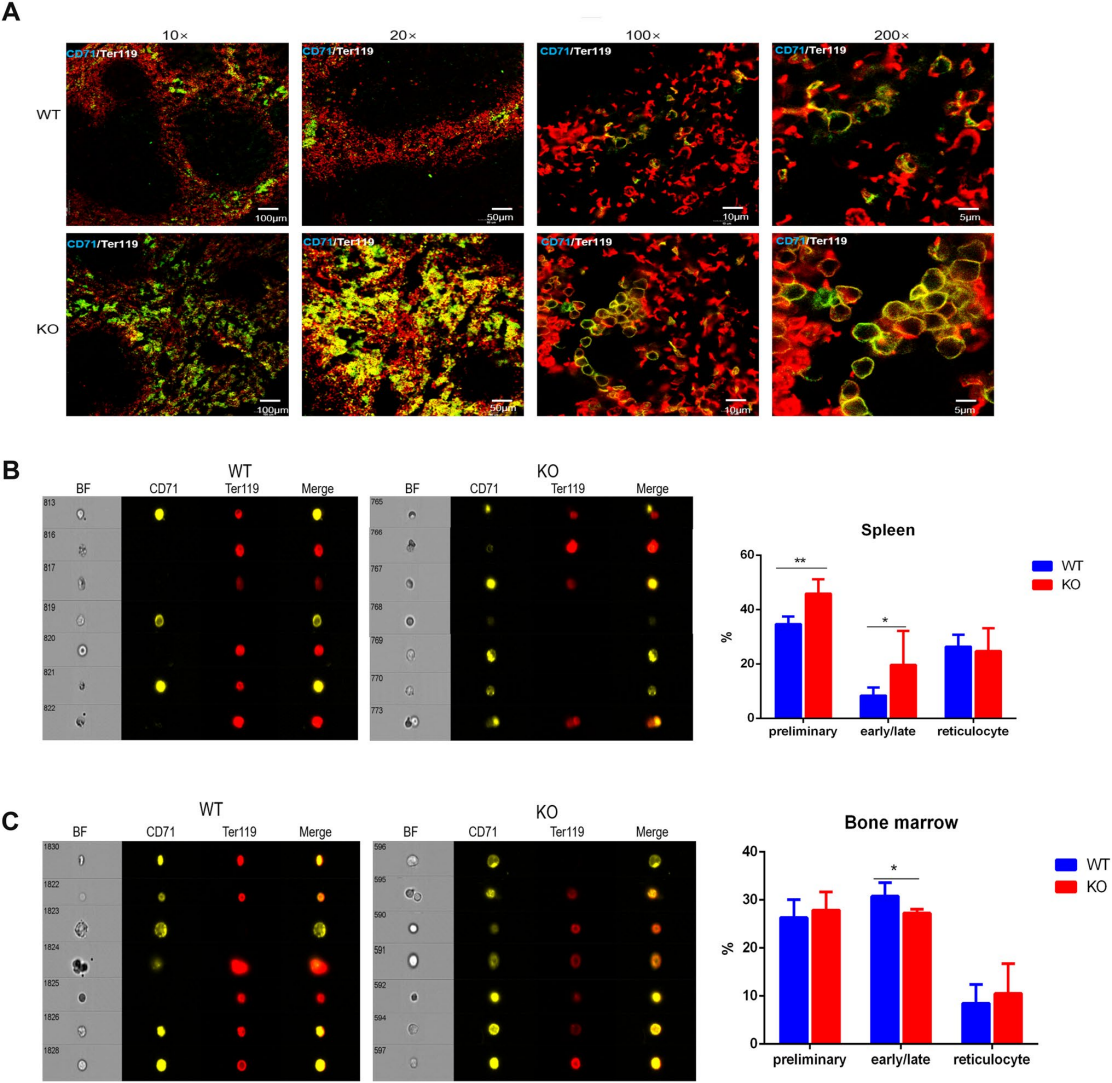
*Zkscan3* is essential for erythropoiesis. **A** Immunofluorescence staining of CD71 (Green) and Ter119 (Red) in spleen sections from Zkscan3^−/−^ and wild-type mice (*n* = 4). **B**–**C** Imaging flow cytometry analysis for CD71 and Ter119 expression in erythroblasts present in spleens and bone marrows of Zkscan3 knockout and wild mice (*n* = 6, replicates per group, non-parametric statistical and Student’s *t* tests). (***p < 0.001, **p < 0.01, *p < 0.05)

Overexpression of *Zkscan3* has been observed in colorectal cancer, breast cancer, bladder cancer, and cervical cancer, suggesting a potential role for *ZKSCAN3* in tumorigenesis (Yang et al. 2008, 2011; Zhang et al. 2012; Chi et al. 2018; Lee et al. 2018). However, *ZKSCAN3* biological functions in a physiological setting are still largely unknown. Therefore, we generated a *Zkscan3* knockout mice model (Fig. 1A). Our previous studies have shown that, in our model, *Zkscan3* genes have been completely knocked out (Zixuan et al. 2019). In this model, we observed a high occurrence (30%) of splenic lesions. The spleen is an important blood filtering and immune organ. Mammalian erythropoiesis follows a defined series of steps leading from the first committed erythroid progenitors to enucleated red blood cells. We analyzed using FACS the percentage of erythrocyte in the four different maturation stages, according to the expression of Ter119 and CD71: preliminary stage (pro-erythroblasts), early-stage (basophilic-erythroblasts), late-stage (chromatophilic-erythroblasts to polychromatophilic-erythroblasts through orthochromatophilic-erythroblasts), and mature stage (reticulocytes). We observed that, in *Zkscan3*^−/−^ mice, splenic erythroblasts in the preliminary, early, and late stages were significantly increased, compared to those in the wild-type mice (Fig. 1B, C). However, the amount of reticulocyte was not statistically different between *Zkscan3*^−/−^ and wild-type mice (Fig. 1B, C). Conversely, in the bone marrow, the number of erythroblasts in the early-stage was significantly decreased in *Zkscan3*^−/−^ mice, whereas late-stage erythroblasts and reticulocytes did not show any obvious difference (Fig. 1D, E). Moreover, we did not find significant differences in the composition of red blood cells, reticulocytes, and MCHC in the peripheral blood of the two groups (Fig. 1F). These data indicated that Zkscan3 may play a role in the differentiation of erythroblasts. Confusingly, the effects in bone marrow and spleen cells are not equivalent. The confocal microscopy and image streaming results also confirmed that the abundance of preliminary stage, early-stage, and late-stage erythroblasts increased in the spleens of the *Zkscan3*^−/−^ mice (Fig. 2A, Green: CD71, Red: Ter119), whereas that of early and late-stage erythroblasts decreased in the bone marrow (Fig. 2C). Here, the abundance of preliminary stage, early-stage and late-stage erythroblasts increased in the spleen of the *Zkscan3*^−/−^ mice and early and late-stage erythroblasts decreased in the bone marrow. This may be because the internal environment of bone marrow and spleen erythroblast growth is different.

### *Zkscan3* is essential for correct erythropoiesis under stress conditions

**Fig. 3:**
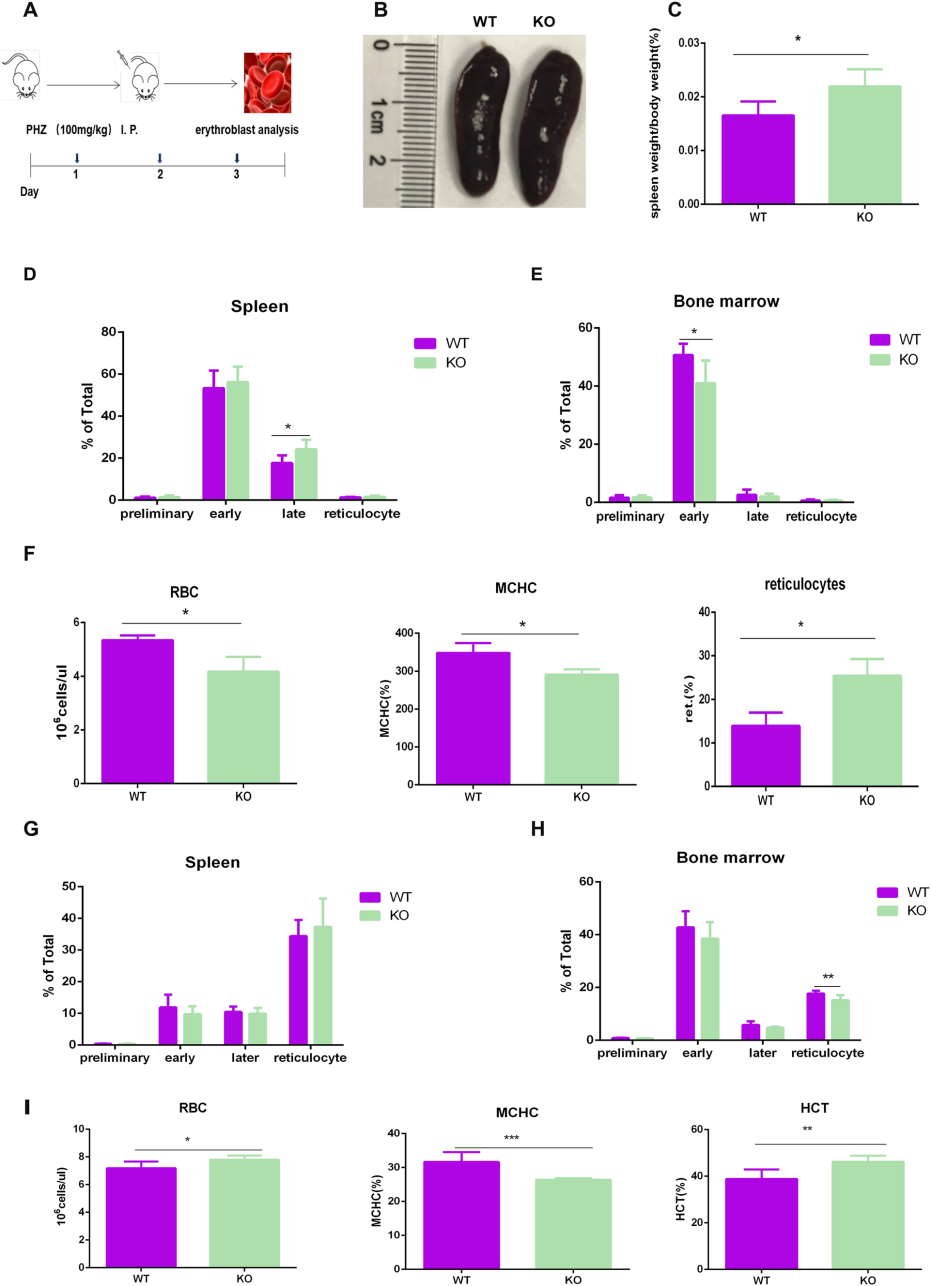
Erythroblast maturation in PHZ-induced *Zkscan3*^−/−^ mice. PHZ (100 mg/kg) was injected into the abdominal cavity of wild-type (WT) and knockout mice (KO). On day 3 and day 10, erythroblasts were detected in bone marrow, spleen, and peripheral blood (*n* = 8, ***p < 0.001, **p < 0.01, *p < 0.05). **A** Experimental scheme. **B** Images of spleen after 3-day treatment in WT and KO mice. **C** Spleen and body weight of mice treated with PHZ on day 3 (*n* = 8, replicates per group, non-parametric statistical and Student’s *t* tests). **D**–**E** Erythroblast subsets in the spleen and bone marrow on day 3 (*n* = 8, replicates per group, non-parametric statistical and Student’s *t* tests). **F** Analysis of peripheral blood (RBC, MCHC and ret.) after 3 days of PHZ treatment (*n* = 8, replicates per group, non-parametric statistical and Student’s *t* tests). **G**–**H** Flow cytometric detection of erythroblasts in the spleen and bone marrow after 10 days of PHZ treatment (*n* = 7, replicates per group, non-parametric statistical and Student’s *t* tests). **I** Analysis of peripheral blood (RBC, MCHC, and HCT) after a 10-day PHZ treatment (*n* = 7, replicates per group, non-parametric statistical and Student’s *t* tests)

PHZ is commonly used to induce hemolytic anemia, through its interaction with hemoglobin (Liao et al. 2018). Therefore, we treated mice with PHZ (Fig. 3A). On day 3, we observed an enlargement of the spleen in *Zkscan3*^−/−^ mice when compared with that in wild-type animals (Fig. 3B). At the same time, the ratio between the spleen percentage and the body weighted was higher in *Zkscan3*^−/−^ mice (Fig. 3C). Moreover, the percentage of late-stage erythroblasts in the spleen significantly increased in *Zkscan3*^−/−^ mice (Fig. 3D), the percentage of early-stage erythroblasts in the bone marrow decreased, and we observed no significant differences for the other erythroblast stages (Fig. 3E). The percentage of reticulocytes increased notably, whereas MCHC and RBCs decreased in the peripheral blood (Fig. 3F). In addition, further analysis revealed that splenic erythrocyte subsets did not change, and the percentage of reticulocytes in the bone marrow decreased (Fig. 3G, H), the number of RBC and HCT increased, and MCHC decreased on day 10 of PHZ treatment (Fig. 3I). These data indicated that erythroblasts in *Zkscan3*^−/−^ mice were more sensitive to PHZ-induced hemolytic anemia, suggesting that *Zkscan3* may play a crucial role in regulating erythropoiesis under stress conditions.

### *Zkscan3*^−/−^ and *Zkscan3*^+/+^ mice display strikingly different expression patterns

**Fig. 4:**
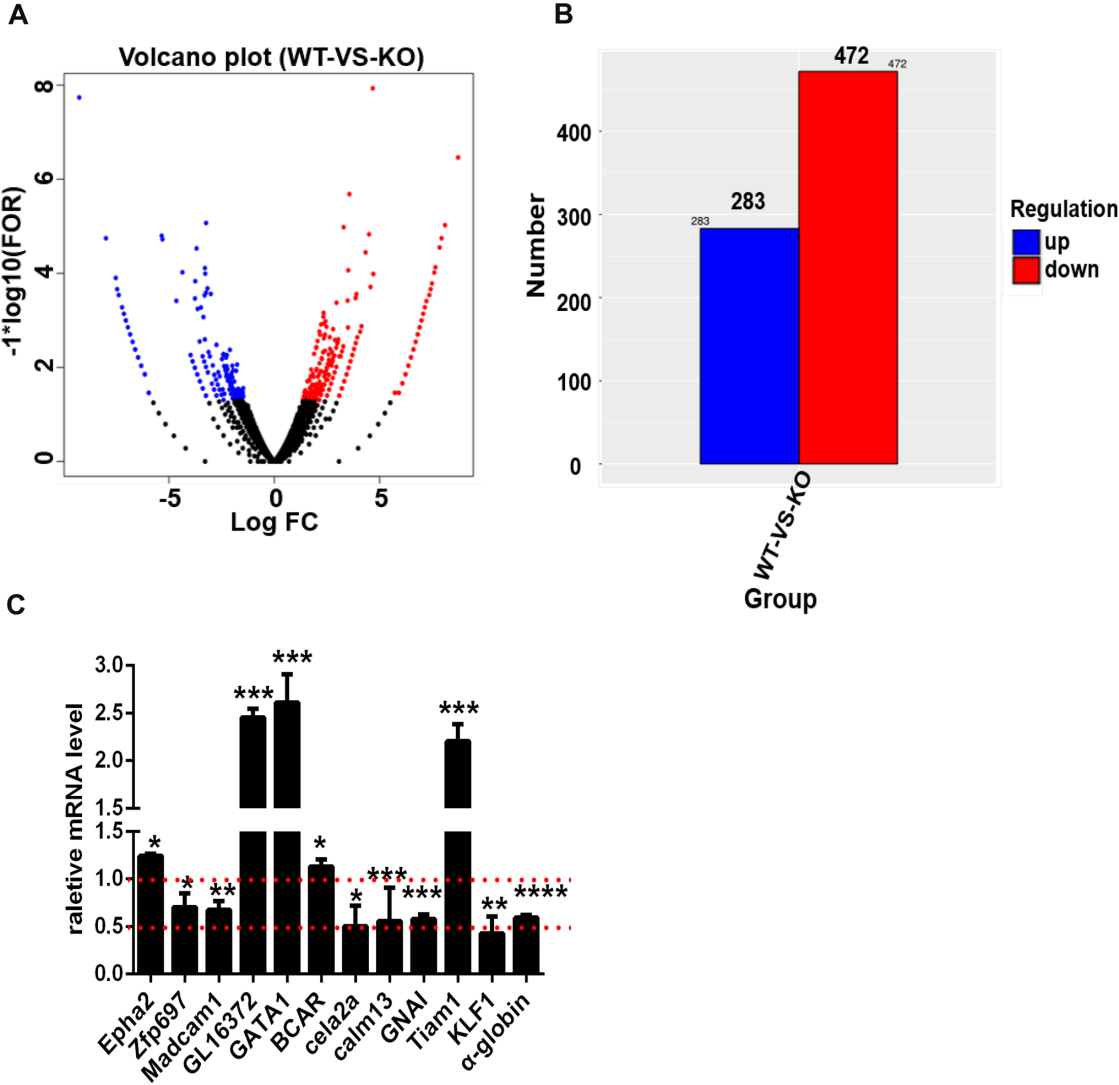
Changes in the expression levels in *Zkscan3*^−/−^ mice. We sorted the early-stage erythroblasts in the spleen of WT and KO mice for RNA-sequence and qPCR analysis (*n* = 4, ***p < 0.001, **p < 0.01, *p < 0.05). **A** Volcano graph of differentially expressed genes. Genes significantly different are indicated by dots: red dots indicate up-regulation and blue dots indicate down-regulation. The x-axis represents the fold change of gene expression in different samples; the y-axis represents the statistical significance of the difference in gene expression (*n* = 4). **B** Sample differences comparing gene expression (*n* = 4). **C** Validation of gene expression in splenic erythroblast by qPCR (*n* = 4, Student’s *t* test)

To better understand how *Zkscan3* regulates erythropoiesis, we performed RNA-sequencing of the early-stage erythroblast (*n* = 3). Our results revealed significant gene expression differences between KO and WT mice (Fig. 4A). Specifically, in *Zkscan3*^−/−^ mice, we found 283 downregulated genes and 472 upregulated genes (Fig. 4B). The differential expression of key genes was validated by qPCR (Fig. 4C). qPCR results confirmed the downregulation of *Madcam1*, *Zfp697*, *Cela2a*, *Calm13*, *Gnai*, *KLF1*, and α-globin (*HBA*). Conversely, the expression of *Epha2*, *Gl16372*, *GATA1*, *Tiam1*, and *Bcar* was upregulated in *Zkscan3*^−/−^ mice. Notably, the degree of upregulation of *GATA1* and *Tiam1*, and the downregulation of *KLF1* was similar (twofold increase *vs*. 50% decrease), compared to those in wild-type mice. It has been reported that GATA1 deficiency is correlated to a lack of erythroblast formation both in vitro and in vivo, which is rescued upon its overexpression (Ling and Crispino 2020). KLF1 is a transcription factor necessary for the enucleation process (Magor et al. 2015; Gnanapragasam et al. 2016). Thus, our results suggest that Zkscan3 may affect the development of erythroblasts by regulating the expression of *GATA1* and *KLF1*.

### Zkscan3 negatively regulates erythroblast development by modulating *GATA1* transcription

**Fig. 5:**
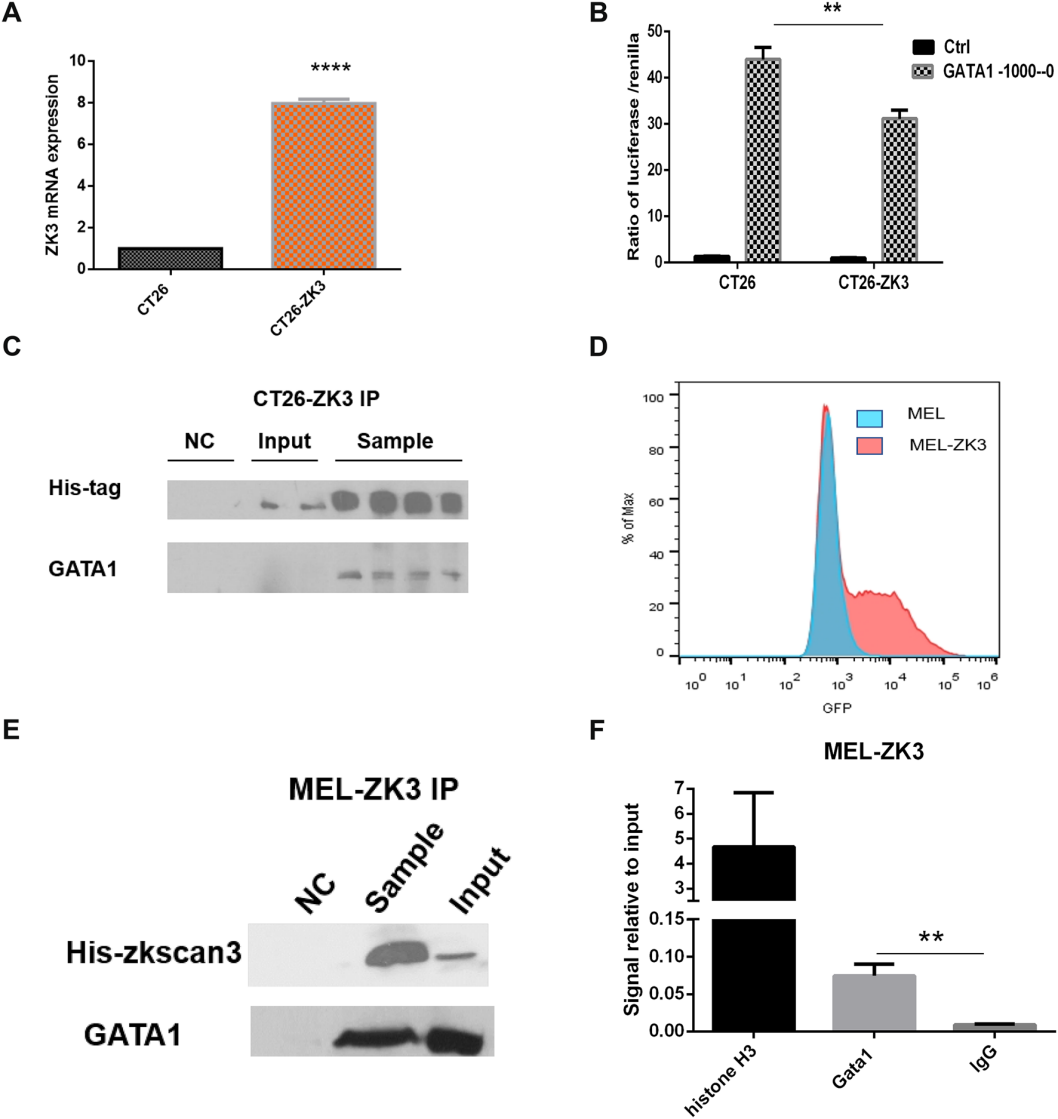
Interaction between Zkscan3 and the *GATA1* promoter. **A** We constructed the Zkscan3-His-GFP fusion insert, and transfected CT26 cells using lentivirus carrying the fusion insert. Quantitative PCR was performed to detect Zkscan3 expression in CT26 cells (*n* = 3, Student’s *t* test). **B** We cloned the − 1000–0 bp Gata1 promoter sequence into the pGL4.17 vector and co-transfected CT26 cells overexpressing Zkscan3 and CT26 cells using the Renilla luciferase plasmid. Luciferase activity was determined 48 h after transfection using a Dual-Luciferase assay kit (Promega, Madison, WI, USA) and measured with a microplate luminometer (Thermo Fisher Scientific). Luciferase reporter assay showing the effect of Zkscan3 on GATA1 promoter activity (*n* = 3, Student’s *t* test). (C) Nuclear extract of CT26-ZK3 cells was used to perform co-immunoprecipitation followed by western blot analysis. (D) We transfected MEL cells with the lentivirus carrying Zkscan3-His-GFP fusion insert, and detected the GFP expression by flow cytometry (*n* = 3, Student’s *t* test). (E) Nuclear extract of MEL-ZK3 cells was used to perform co-immunoprecipitation followed by western blot analysis. For detailed experimental procedures, please see “Materials and methods” section. **F** Chromatin immunoprecipitation (ChIP) of *GATA1* promoter with ZKSCAN3 in *Zkscan3-*overexpressing MEL cells (MEL-Zkscan3). His-tagged ZKSCAN3 protein was pulled down using a specific anti-His antibody. To detect the binding of ZKSCAN3 to the *GATA1* promoter, cross-linked DNA was amplified using quantitative PCR and specific primers. The positive control and the negative control antibodies and primers were provided in the kit. All experiments were performed according to manufacturer’s instructions (catalog number: 56383, Cell Signaling Technology) (*n* = 3, Student’s *t* test). (***p < 0.001, **p < 0.01, *p < 0.05)

Since *Zkscan3* knockout was associated with the up-regulation of GATA1, we hypothesized that Zkscan3 may affect the development of erythroblasts by directly regulating the transcription activity of GATA1. To test this hypothesis, we tested if Zkscan3 interacted with the GATA1 promoter. First, we generated CT26 cells overexpressing *Zkscan3* (Fig. 5A). We observed that the luciferase/Renilla signal was weakened in CT26-ZK3 cells compared with wild-type CT26 cells when we transfected the plasmid containing the GATA1 promoter (Fig. 5B). It suggested that Zkscan3 may bind to GATA1 promoter, and suppress GATA1 expression. However, the expression level of endogenous GATA1 in CT26 was relatively low (Fig. 5C), thus we use MEL cells to further verify the relationship between zkscan3 and the promoter of *GATA1*. To test whether Zkscan3 and *GATA1* promoter interacted, we overexpressed *Zkscan3* in MEL cells (Fig. 5D), and performed co-immunoprecipitation followed by western blot analysis using MEL-ZK3 cell line overexpressing *Zkscan3*. Results show that *GATA1* promoter and Zkscan3 directly interact with each other (Fig. 5E). Next, we performed chromatin immunoprecipitation (ChIP) to examine the interaction between Zkscan3 and *GATA1* promoter (Fig. 5F). Results further confirmed that Zkscan3 binds to the promoter of *GATA1*.

### Zkscan3 regulates erythroblast development by modulating KLF1 expression

**Fig. 6:**
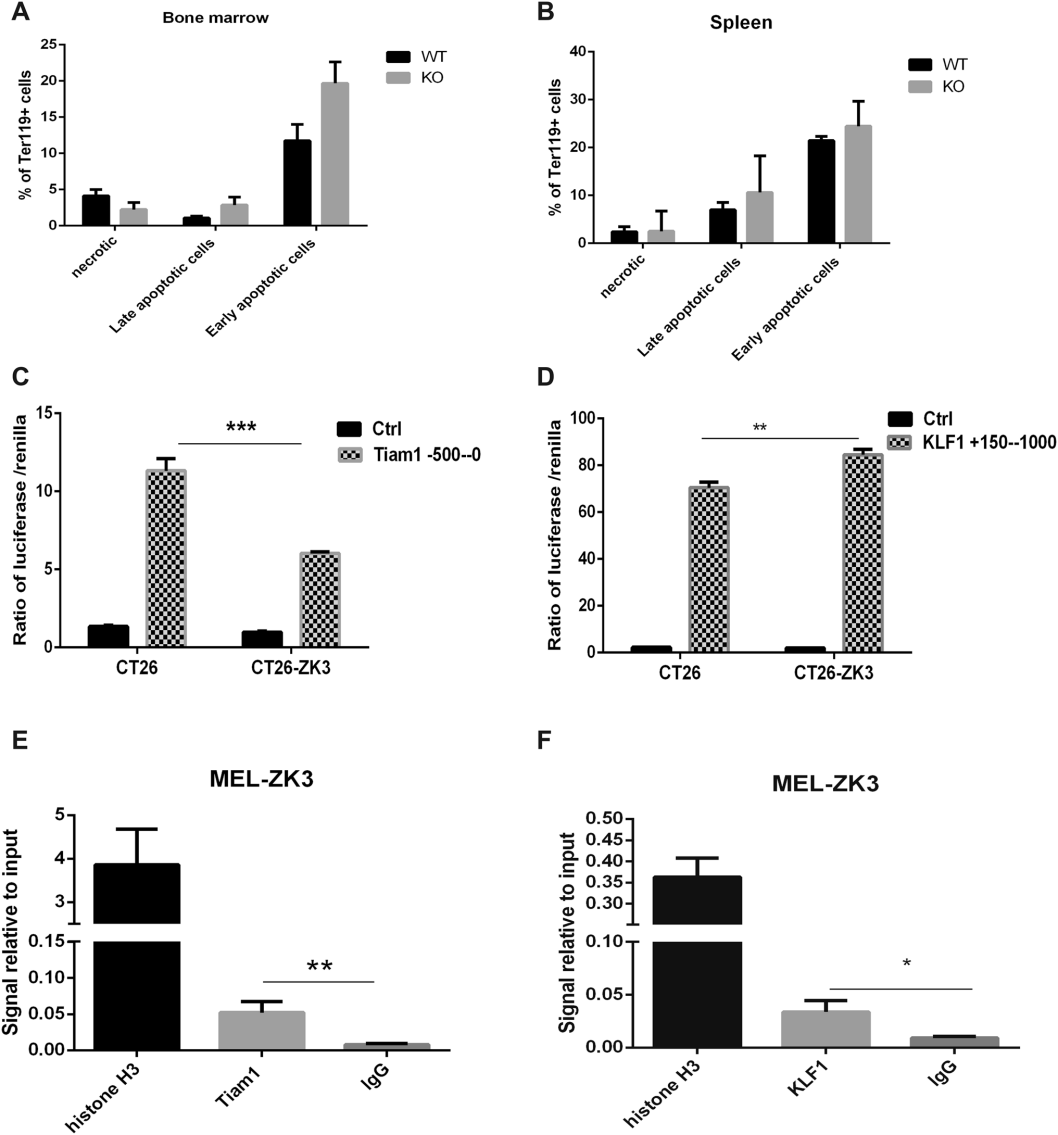
ZKSCAN3 interacts with the promoter of KLF1 and Tiam1. **A**–**B** We selected 6–8-weeks-old wild-type and Zkscan3 knockout mice, and stained with annexin-V/7-AAD and Ter119 for flow cytometric analysis. Apoptotic erythroblast in the bone marrow and in the spleen of WT and KO mice (*n* = 8, replicates per group, non-parametric statistical and Student’s *t* tests). **C**–**D** We cloned the − 1000–0 bp KLF1 and − 500–0 bp Tiam1 promoter sequence into the pGL4.17 vector and co-transfected CT26 cells overexpressing *Zkscan3* and CT26 cells with the Renilla luciferase plasmid. Luciferase activity was determined 48 h after transfection using a Dual-Luciferase assay kit and a microplate luminometer. Luciferase reporters assay showing the effect of ZKSCAN3 on *KLF1* and *Tiam1* promoter activity (*n* = 3, Student’s *t* test). **E**–**F** The interaction between Zkscan3 and the promoters of *Tiam1* and *KLF1* was confirmed by ChIP using MEL-ZK3 cells (*n* = 3, Student’s *t* test). His-tagged ZKSCAN3 protein was pulled down using a specific anti-His antibody. To detect the ZKSCAN3 binding to the *KLF1* and *Tiam1* promoters, cross-linked DNA was subjected to qPCR using specific primers; the positive and the negative control antibodies and primers were provided in the kit. All experiments were carried out in accordance with manufacturer’s instructions. (***p < 0.001, **p < 0.01, *p < 0.05)

The erythroblast maturation phenotype in *Zkscan3*^−/−^ mice suggests that in this genetic background erythroblasts may have undergone either apoptosis or an enucleation disorder. To verify this hypothesis, we stained erythroblast with Annexin V-APC/7-AAD to detect apoptosis. Then, we performed flow cytometry and observed that *Zkscan3*^−/−^ mice had no significantly apoptotic erythroblasts in both the bone marrow and the spleen than wild-type mice (Fig. 6A, B). Previous studies have shown that Tiam1 is involved in the remodeling of the cytoskeleton (Payapilly et al. 2021) and increased expression of Tiam1 can cause DNA damage and lead to cell apoptosis (Zhu et al. 2014). Regarding the enucleation disorder, KLF1 is known to affect erythroblast enucleation by regulating the expression of cell cycle proteins, deacetylases, caspases, and nuclear membrane proteins (Magor et al. 2015; Gnanapragasam et al. 2016). Since both of these genes were differentially expressed in *Zkscan3*^−/−^ mice (Fig. 4A–C), we tested whether Zkscan3 could interact with *Tiam1* and *KLF1* promoters. The luciferase/Renilla signal was decreased in CT26-ZK3 cells transfected with the plasmid containing the Tiam1 promoter and was increased in cells containing the *KLF1* promoter (Fig. 6C, D). The interactions between Zkscan3 and promoters of *Tiam1* and *KLF1* was further confirmed by ChIP (Fig. 6E, F). Based on our results, we concluded that Zkscan3 binds to the promoters of *Tiam1* and *KLF1*.

## Discussion

The role of Zkscan3 in tumor pathogenesis is well known (Yang et al. 2008, 2011; Zhang et al. 2012; Chi et al. 2018; Lee et al. 2018). However, the role of Zkscan3 under physiological conditions, especially in the context of erythroblast development, is poorly understood.

In this study, we reported that in a murine *Zkscan3* knockout model, the percentage of early-stage and late-stage erythroblasts in the spleen and the percentage of early-stage erythroblast in the bone marrow decreased, despite the number of reticulocytes did not change. We assume that it may be due to the compensatory regulation of the mouse hematopoietic system. We also studied stress erythropoiesis by treating mice with PHZ. Following PHZ treatment, the number of early-stage and late-stage erythroblasts in the spleen of *Zkscan3*^−/−^ mice increased significantly and the number of early-stage erythroblast in the bone marrow decreased remarkably. Moreover, the percentage of reticulocyte significantly increased, whereas MCHC and RBC decreased. The difference observed between the spleen and bone marrow can be explained by the inherently different stress erythropoiesis process between these two organs (Kalfa et al. 2010). It is also worth noting that Zkscan3 knockout may cause stress erythropoiesis, contributing to the difference observed between the spleen and the bone marrow. Taken together, our experimental results demonstrate for the first time the key role of Zkscan3 in erythroblast development.

Normal erythroblast differentiation is controlled by erythropoietin and inherent multimeric transcription complexes, including GATA1 complexes, that can activate or inhibit the transcription of erythropoiesis target genes (Kerenyi and Orkin 2010; Kuhrt and Wojchowski 2015; Valent et al. 2018). The differentiation of terminal erythrocyte is also regulated by KLF1, which can bind to DNA next to the GATA1 complex to jointly regulate erythroid genes (Gnanapragasam et al. 2016). Therefore, *GATA1* and *KLF1* mutations are associated with altered erythropoiesis (Singleton et al. 2012; Doshi et al. 2014). As high Zkscan3 expression has been reported previously in colorectal cancer cells, we therefore evaluated CT26 cells and found that are Zkscan3-positive too. Accordingly, we selected CT26 cells for luciferase assay and co-IP assay, as well as a MEL cell line a to examine the interaction between Zkscan3 and *GATA1*, *KLF1*, and *Tiam1* promoters. In this study, we reported that the expression of *GATA1* is upregulated in Zkscan3^−/−^ erythroblasts and that Zkscan3 can bind to the *GATA1* promoter, and directly to GATA1, to inhibit its activity. Consistently, our results demonstrate that the expression of *KLF1* was reduced in *Zkscan3*^−/−^ mice and that Zkscan3 can bind to *KLF1* promoter. However, further investigation is required to clarify this pathway, since *Zkscan3*^−/−^ mice display the same number of reticulocytes as wild-type mice. *Tiam1* expression was significantly increased upon *Zkscan3* knockout and Zkscan3 was found to bind *Tam1* promoter both in vitro and in vivo. Tiam1 is a Rac1-specific guanine nucleotide exchange factor that selectively activates Rac1 (Yue et al. 2021). It has been reported that Tiam1 participates in cytoskeleton rearrangement, cell migration, and mobility (Zhu et al. 2014; Izumi et al. 2019; Payapilly et al. 2021). However, whether Tiam1 affects erythroblast development remains to be further determined.

Interestingly, GATA1 has been reported to have antiapoptotic functions (Aguirre et al. 2010; Juban et al. 2021). In current manuscript, we did not find statistical difference of erythroblast apoptosis between wild-type and knockout mice in the bone marrow and spleen (Fig. 6A, B) although upregulation of GATA1 in our knockout mice were observed. This could be partially explained by upregulation of Tiam1 in knockout mice because Tiam1 has been suggested to play a proapoptotic role (Zhu et al. 2014). Consequently, the apoptosis degree in erythropoiesis between wild-type and knockout mice were not significantly different. The limitation is that we still do not know the specific binding sites of Zkscan3 and *GATA1*, *KLF1*, and *Tiam1* promoters, which will be the focus on our future research.

In conclusion, we provide here a mechanistic explanation for the abnormal erythroblast development observed upon Zkscan3 deletion, describing novel interactions between Zkscan3, GATA1, KLF1, and Tiam1.
